# COVID-19 vaccine acceptance and its determinants among Vietnamese teachers: a web-based cross-sectional survey

**DOI:** 10.3934/publichealth.2022042

**Published:** 2022-07-25

**Authors:** Anh Thi-Kim Le, Thanh Quoc Pham, Long Thanh Nguyen, Tien Dung Pham, Nhu Van Ha

**Affiliations:** 1 Department of Information system, Hanoi University of Public Health, 1A Duc Thang Road, Bac Tu Liem District, Hanoi, Vietnam; 2 Center for Injury Policy and Prevention Research, Hanoi University of Public Health, 1A Duc Thang Road, Bac Tu Liem District, Hanoi, Vietnam; 3 Department of Nursing, National Hospital of Dermatology and Venereology, 15A Phuong Mai, Dong Da District, Hanoi, Vietnam; 4 Faculty of Clinical Medicine, Hanoi University of Public Health, 1A Duc Thang Road, Bac Tu Liem District, Hanoi, Vietnam

**Keywords:** COVID-19, vaccine acceptance, determinants, teachers, Vietnam

## Abstract

**Objectives:**

This paper aimed to describe acceptance of the COVID-19 vaccine and its determinants among Vietnamese teachers.

**Methods:**

This was a web-based cross-sectional survey with a sample of 17,176 teachers from kindergarten to high school who currently reside in Vietnam. A participant who exhibited “acceptance” towards the vaccine gave the following response: *“have the readiness to get COVID-19 vaccine”*.

**Results:**

About 88% of all participants were accepting of the COVID-19 vaccine, while 70.4% were willing to pay (WTP) for it. The vaccine acceptance rate increased by age with odds ratios (OR) of 1.65 (1.41–1.93), 1.96 (1.67–2.29), and 2.4 (1.95–2.95) for the age groups 30–39, 40–49, and 50–59 respectively, when compared to the 18–29 age group. Male were found to be more likely to accept the vaccination than females (OR = 1.16; 95% CI: 1.02–1.31); teachers without a chronic disease were 4.13 times (95% CI: 2.67–6.37) more likely to accept the vaccine than those with an underlying condition. Willingness to pay and beliefs about the safety and efficacy of the vaccine were major factors in driving participants' responses.

**Conclusion:**

A high proportion of COVID-19 vaccine acceptance is a promising indicator of high coverage among this priority group for vaccination. Communication campaigns should consider addressing determinants uncovered by this study to achieve better vaccine acceptance.

## 1. Introduction

The COVID-19 pandemic (caused by SARS-CoV-2 virus) started at the end of 2019 and has resulted in 180 million cases and 3.9 million of deaths worldwide as of June 2021 [1]. Many countries have applied non-pharmaceutical prevention mechanisms such as the regular use of face masks, maintenance of physical distance, avoidance of public areas, sanitization of the hands, restriction of travel, and the closure of schools to decrease virus transmission [2],[3]. These interventions have had some success in reducing the pandemic's burden on medicine and global economics, but could not wholly alleviate the strain. While the efficacy of antiviral drugs has been questioned heavily in recent years, vaccination remains the most effective response to the pandemic [4],[5].

At the time of this study, the World Health Organization (WHO) has listed Pfizer/BioNTech, Astrazeneca-SK Bio, Serum Institute of India, Janssen, Moderna, and Sinopharm vaccines as acceptable emergency use vaccines [6]. At present, Vietnam only has the Astrazeneca-SK Bio vaccine through COVAX and the support of the Japanese government. Due to the limited number of COVID-19 vaccine doses, the Decision 1210/QD-BYT of the Vietnam Ministry of Health (MoH) listed priority groups for vaccination. These priority groups change over time; for example, the priority groups during the first quarter of 2021 were healthcare workers and first responders. The following quarter prioritized diplomats, customs officials, immigration workers, military and police force, and teachers [7].

The vaccines approved by WHO and the Food and Drug Administration (FDA) in the United States (US) are showing good efficacy in combating SARS-CoV-2, but the proportion of vaccination refusal remains high worldwide [8]. For example, this proportion was 24% in France, 10% in Germany (an additional 20% reported uncertainty as to whether or not they would get the vaccine), and 13% in Australia [8]. In Vietnam, a recent WHO/WPRO report in May 2021 indicated that about 3% of Vietnamese would not get the COVID-19 vaccine and 28% would wait [9]. Although the proportion of participant refusal of the COVID-19 vaccination among Vietnamese in this survey is less than that of other countries, there is still a large proportion of people who wish to temporarily delay their vaccination. Evidence indicates that if vaccine acceptance is low, vaccination will be insufficient to reach herd immunity, which is a key control factor of the COVID-19 pandemic [10].

As mentioned above, teachers are a priority group for vaccination in Vietnam. The vaccination coverage of this group would contribute to the requirements for herd immunity; at the same time, vaccinating teachers would impact parents' choices in sending their children back to school. There is no data highlighting teachers' resistance to vaccination; however, this proportion can be assumed to be consistent with the population average of 3%. In order to maximize vaccination rates amongst teachers, there is a need to uncover social determinants for vaccine resistance in this particular demographic. Therefore, this paper is aimed to be the first study in Vietnam to detail COVID-19 vaccine acceptance and its determinants among Vietnamese teachers.

## 2. Materials and methods

### 2.1. Study design and participant

This study was conducted via a web-based cross-sectional survey. Study participants were kindergarten to high school teachers aged 18 and older who were Vietnamese residents at the time the study was conducted. Participants were required to have an internet connection. Participation in the study was voluntary.

### 2.2. Sample sizes, sampling and data collection

We aimed to identify social determinants of vaccine resistance with relation to the age of a teacher's students; the four following groups were formed for this analysis: kindergarten teachers, primary school teachers, secondary school teachers, and high school teachers. We assumed that the proportion of COVID-19 vaccine resistance would be low (<5%); to account for this we calculated a sample size to estimate this proportion with 10% of relative precision and 2 of design effect. As a result, the total sample size needed was about 14,600 teachers.

Data collection began on June 1, 2021 and closed on June 10, 2021. Given pandemic-related limitations, the survey was conducted online. With around 65 million social media users in Vietnam in January 2020, the study was expected to reach many demographics, so a convenience sample approach was applied. Operating on the social media platforms Facebook and Zalo, researchers sent an invitation letter explaining the purpose of the study, the process of the study, ethical concerns, and an online questionnaire. The first respondents were teachers known by the research team members and were asked to share the invitation link to as many other colleagues and academic professionals. As survey participation was voluntary, we didn't include a consent form. A total of 17,176 participants completed the questionnaire.

### 2.3. Survey instrument and expected outcomes

The study questionnaire consisted of 4 parts: demographic information, acceptance of COVID-19 vaccination, and beliefs in and concerns about COVID-19 vaccines.

The focal question of this survey was the one regarding COVID-19 vaccine acceptance. A participant was seen as “accepting” if he/she responded “yes” for the question “Do you have the readiness to get COVID-19 vaccine?”. If the answer was “No” or “No decision at this time”, the participant was seen as “not accepting”. No name of vaccine was specified.

One of the independent variables included a participant's willingness to pay (WTP) for the vaccine. This variable was assessed by the question “Do you have the willingness to pay for your COVID-19 vaccine?”. If the answer was “yes”, a participant was seen as “WTP”; if “no”, a participant was seen as “no WTP”.

Four questions regarding belief in COVID-19 vaccine were displayed in the 5-Likert scale from 1 “strongly disagree” to 4 “strongly agree”, with an option 5 of “don't know/no response”. These responses were coded to reflect “agreement” for answers 3 and 4 and “disagreement” for answers 1, 2, and 5. These 4 questions were developed using the Health Belief Model from a study by Zampetakis and Melas [11]. Seven questions regarding concerns about the vaccine revealed each respondent's level of prior knowledge about the vaccine. We referred to a study on information-seeking behaviors to develop these 7 questions [12].

### 2.4. Data analysis

Frequencies and proportions were used to describe different characteristics of study participants' beliefs, concerns, and acceptance of the vaccine. Multivariate logistic regression was used to assess determinants of vaccine acceptance. All independent variables that had statistically significant relation to research outcome were added into the multivariate model by the Enter method. The final model accepted independent variables with a p-value of less than 0.2 and the fit of the final model was confirmed through p-value references from the Hosmer-Leme show test and the normal distribution of residual.

### 2.5. Ethical concerns

The study was approved by the Ethical Committee of HUPH (Ref. 204/2021/YTCC-HD3, dated May 6, 2021). All relevant ethical issues were assessed and monitored by the research team throughout the completion of the study. As previously stated, no consent form was required. Personal information such as name and home or school address were not required to be provided.

## 3. Results

Our study received 17,209 responses, of which 17,176 were completed and included in the final analysis. The number of kindergarten, primary, secondary and high school teachers was 4733 (27.5%) 5959 (34.7%), 3738 (21.8%), and 2746 (16.0%), respectively. More than half of respondents (53.4%) were living in a city or town. Over two-third of respondents (74.5%) were between 30 and 49 years of age. Female teachers accounted for about 75% of all participants. The proportion of female teachers was higher than male teachers at all grade levels. This finding was particularly pronounced amongst kindergarten teachers (88.2% female). Nearly 90% of teachers were married; a similar proportion was noted with regard to being non-religious. 74.7% of the teachers were of the Kinh ethnic group (the majority group in Vietnam), and the majority teachers (95.7%) had not completed graduate education.

Table 1 shows that the large majority of teachers (88%) accept the vaccine. The highest acceptance rate was found in primary school teachers (89.4%) and the lowest in high school teachers (85.5%). About 70% of respondents were willing to pay a vaccination fee; teachers at high schools had the highest WTP (72%) while teachers of kindergarten students had the lowest (69.7%). However, the difference in WTP across grade levels was not significant. Moreover, about 95% of teachers expressed being prepared to take the Vietnamese vaccine.

**Table 1.** Demographic characteristics and acceptance for COVID-19 vaccination among study participants.

Factor	Kindergarten	Primary school	Secondary school	High school	Total
n	4733	5959	3738	2746	17,176
	n (%)	n (%)	n (%)	n (%)	n (%)
***Demographic characteristics***					
Living place					
City/town	2254 (47.6)	3194 (53.6)	2121 (56.7)	1600 (58.3)	9169 (53.4)
Mountainous area	947 (20.0)	1803 (30.3)	1012 (27.1)	820 (29.9)	4582 (26.7)
Countryside	1532 (32.4)	962 (16.1)	605 (16.2)	326 (11.9)	3425 (19.9)
Age group					
18–29	925 (19.5)	722 (12.1)	203 (5.4)	129 (4.7)	1979 (11.5)
30–39	2223 (47.0)	1408 (23.6)	1191 (31.9)	1101 (40.1)	5923 (34.5)
40–49	1211 (25.6)	2540 (42.6)	1857 (49.7)	1309 (47.7)	6917 (40.3)
50–59	374 (7.9)	1289 (21.6)	487 (13.0)	207 (7.5)	2357 (13.7)
Gender					
Female	4176 (88.2)	4450 (74.7)	2570 (68.8)	1742 (63.4)	12,938 (75.3)
Male	557 (11.8)	1509 (25.3)	1168 (31.2)	1004 (36.6)	4238 (24.7)
Marital status					
Single	276 (5.8)	264 (4.4)	104 (2.8)	125 (4.6)	769 (4.5)
Married	4167 (88.0)	5263 (88.3)	3422 (91.5)	2466 (89.8)	15,318 (89.2)
Divorced/widowed	290 (6.1)	432 (7.2)	212 (5.7)	155 (5.6)	1089 (6.3)
Ethnic					
Kinh	3061 (64.7)	4522 (75.9)	2950 (78.9)	2292 (83.5)	12,825 (74.7)
Other	1672 (35.3)	1437 (24.1)	788 (21.1)	454 (16.5)	4351 (25.3)
Religion					
None	4164 (88.0)	5475 (91.9)	3395 (90.8)	2524 (91.9)	15,558 (90.6)
Yes	569 (12.0)	484 (8.1)	343 (9.2)	222 (8.1)	1618 (9.4)
Education					
Graduate	46 (1.0)	60 (1.0)	95 (2.5)	540 (19.7)	741 (4.3)
Undergraduate & below	4687 (99.0)	5899 (99.0)	3643 (97.5)	2206 (80.3)	16,435 (95.7)
***Acceptance for COVID-19 vaccination***					
COVID-19 vaccine acceptance					
No	35 (0.7)	26 (0.4)	29 (0.8)	23 (0.8)	113 (0.7)
Yes	4187 (88.5)	5326 (89.4)	3250 (86.9)	2349 (85.5)	15,112 (88.0)
Not yet	511 (10.8)	607 (10.2)	459 (12.3)	374 (13.6)	1951 (11.4)
Ready to pay vaccination fee					
No	257 (5.4)	376 (6.3)	163 (4.4)	99 (3.6)	895 (5.2)
Yes	3300 (69.7)	4186 (70.2)	2632 (70.4)	1976 (72.0)	12,094 (70.4)
Not yet	1176 (24.8)	1397 (23.4)	943 (25.2)	671 (24.4)	4187 (24.4)
Readiness of getting Vietnamese vaccines					
Agree	3923 (97.9)	4714 (96.7)	2743 (95.9)	1914 (93.9)	13,294 (96.5)
Disagree	86 (2.1)	160 (3.3)	118 (4.1)	124 (6.1)	488 (3.5)

**Table 2.** Belief of study participants in COVID-19 vaccine.

Factor	Kindergarten	Primary school	Secondary school	High school	Total
n	4733	5959	3738	2746	17,176
	n (%)	n (%)	n (%)	n (%)	n (%)
***Beliefs in COVID-19 vaccine***					
Vaccines are safe
Agree	3944 (97.9)	4923 (97.9)	2995 (97.2)	2139 (95.6)	14,001 (97.4)
Disagree	86 (2.1)	106 (2.1)	86 (2.8)	98 (4.4)	376 (2.6)
Vaccination can protect people from SARS-CoV-2					
Agree	4121 (97.8)	5232 (97.9)	3257 (97.2)	2337 (96.3)	14,947 (97.4)
Disagree	93 (2.2)	114 (2.1)	94 (2.8)	91 (3.7)	392 (2.6)
The more people vaccinated, the quicker pandemic stopped					
Agree	4398 (99.1)	5648 (99.5)	3531 (99.2)	2591 (99.0)	16,168 (99.2)
Disagree	42 (0.9)	31 (0.5)	30 (0.8)	26 (1.0)	129 (0.8)
Vaccines from the US and Europe are better than others					
Agree	2347 (85.0)	3032 (82.5)	1849 (82.3)	1478 (83.6)	8706 (83.3)
Disagree	415 (15.0)	641 (17.5)	399 (17.7)	290 (16.4)	1745 (16.7)
***Concerns of study participants***					
Information searching about COVID-19 vaccine					
No	64 (1.4)	72 (1.2)	50 (1.3)	53 (1.9)	239 (1.4)
Yes	4669 (98.6)	5887 (98.8)	3688 (98.7)	2693 (98.1)	16,937 (98.6)
Safety of vaccine
No	860 (18.2)	719 (12.1)	402 (10.8)	241 (8.8)	2222 (12.9)
Yes	3873 (81.8)	5240 (87.9)	3336 (89.2)	2505 (91.2)	14,954 (87.1)
Effectiveness of vaccine
No	2200 (46.5)	1872 (31.4)	1073 (28.7)	787 (28.7)	5932 (34.5)
Yes	2533 (53.5)	4087 (68.6)	2665 (71.3)	1959 (71.3)	11,244 (65.5)
Post-injection adverse events
No	2803 (59.2)	2571 (43.1)	1459 (39.0)	981 (35.7)	7814 (45.5)
Yes	1930 (40.8)	3388 (56.9)	2279 (61.0)	1765 (64.3)	9362 (54.5)
Vaccine cost
No	3670 (77.5)	4063 (68.2)	2529 (67.7)	1853 (67.5)	12,115 (70.5)
Yes	1063 (22.5)	1896 (31.8)	1209 (32.3)	893 (32.5)	5061 (29.5)
Place to be vaccinated
No	3508 (74.1)	3561 (59.8)	2143 (57.3)	1543 (56.2)	10,755 (62.6)
Yes	1225 (25.9)	2398 (40.2)	1595 (42.7)	1203 (43.8)	6421 (37.4)
Vaccine expiration date
No	3335 (70.5)	3246 (54.5)	2074 (55.5)	1494 (54.4)	10,149 (59.1)
Yes	1398 (29.5)	2713 (45.5)	1664 (44.5)	1252 (45.6)	7027 (40.9)
Country made
No	3523 (74.4)	3520 (59.1)	2040 (54.6)	1437 (52.3)	10,520 (61.2)
Yes	1210 (25.6)	2439 (40.9)	1698 (45.4)	1309 (47.7)	6656 (38.8)

Nearly 100% of teachers believed that COVID-19 vaccines are not only safe, but efficacious in controlling the virus. Similarly, almost all teachers agreed that the more people get the COVID-19 vaccine, the faster the pandemic will end. We didn't distinguish between different COVID-19 vaccine brand names in the question “Do you believe that the COVID-19 vaccines imported from the United States or Europe are better than those from other countries?” Over 80% of respondents agreed with this question (Table 2).

As shown in Table 2, almost all teachers paid attention to vaccine-related updates and information (98.6%). Most of their concern was focused on the safety of vaccines, with 81.7% respondents selecting this answer. High school teachers were the most concerned about vaccine safety, while kindergarten teachers were the least. Respondents displayed considerably lower regard for other factors, such as vaccine effectiveness (65.5% of respondents reported concern), side effects (54.5%), expiration date (40.9%), country of origin (38.8%), and cost (29.5%). For every factor studied, teachers at the high school level always showed the greatest level of concern when compared to teachers at other levels.

**Figure 1.**
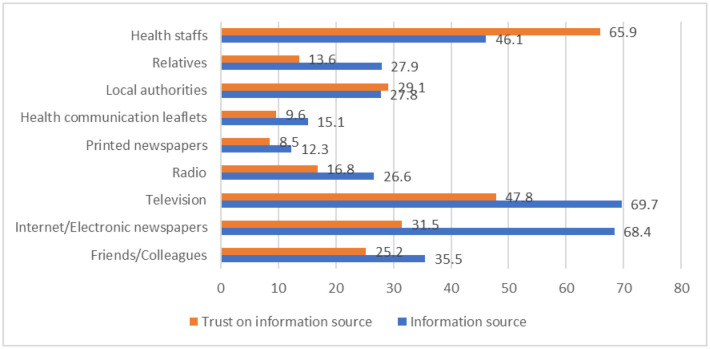
The proportion of COVID-19 vaccine information sources and trust in these sources.

Our results indicated that respondents received news and information pertaining to the pandemic from a host of different sources. The two leading information sources were television (69.7% of respondents relied on this source) and internet/electronic newspapers (68.4%). However, nearly two third of all respondents (65.9%) reported healthcare professionals as the most reliable information source, followed by television (47.8%). Printed newspapers (8.5%) and health communication leaflets (9.6%) were the least trusted information sources (Figure 1).

**Table 3.** Determinants of COVID-19 vaccine acceptance among study participants.

	OR	95% CI	P
**Age group,** *Ref: 18–29*
30–39	1.65***	[1.41,1.93]	<0.0001
40–49	1.96***	[1.67,2.29]	<0.0001
50–59	2.40***	[1.95,2.95]	<0.0001
**Gender,** *Ref: Female*	1.16*	[1.02,1.31]	0.019
**Having a chronic disease,** *Ref: Yes*	4.13***	[2.67,6.37]	<0.0001
**Not trust any information,** *Ref: Yes*	7.50***	[3.57,15.74]	<0.0001
**Belief in safety of the vaccine,** *Ref: Agree*
Disagree	0.14***	[0.11,0.18]	<0.0001
Don't know	0.21***	[0.19,0.24]	<0.0001
**Beliefs in capacity of pandemic control of vaccine** *Ref: Agree*
Disagree	0.37***	[0.24,0.57]	<0.0001
Don't know	0.40***	[0.33,0.47]	<0.0001
**WTP for COVID-19 vaccines,** *Ref: No*
Yes	1.62***	[1.30,2.01]	<0.0001
Not decided	0.47***	[0.38,0.59]	<0.0001

*Note: N= 17,176; p = 0.19 (Hosmer and Lemeshow test).

Table 3 presents the results of multivariate logistic regression to identify determinants of COVID-19 vaccine acceptance among Vietnamese teachers. Our results indicated that teachers aged above 30 (30–39, 40–49, and 50–59) were more likely to accept the COVID-19 vaccination than teachers aged from 18 to 29 years old (OR = 1.65, 95% CI: 1.41,1.93; OR = 1.96, 95% CI: 1.67,2.29; and OR = 2.4, 95% CI: 1.95,2.95, respectively). Teachers, who did not have any chronic diseases were 4.13 times (95% CI: 2.67,6.37) more likely to accept the vaccination than teachers with any chronic disease. Compared to teachers who believed in the safety and efficacy of COVID-19 vaccines, teachers without these beliefs were naturally less likely to accept a vaccine. Also, teachers who had WTP for the vaccination were more likely to accept the vaccination than those who had reluctant or unclear WTP statuses.

## 4. Discussion

### 4.1. What is already known on the topic and what this study adds

The most striking finding in our survey was the high proportion of study participants who accepted the COVID-19 vaccine (88%). This percentage of acceptance is higher than that in most countries according to the review of Eric Robinson, Andrew Jones, India Lesser, and Michael Daly (2021) [8]. The countries with the largest percentage of citizens who accept the vaccine are the United Kingdom (94%), Ireland (91%), and China (91%), where having their own vaccine listed by WHO. Many of the other reports were not consistent with these three nations, showing that a number of countries' citizens were resistant to vaccination. It must be noted that, while our study focused solely on the teacher demographic, reports from other countries were more nationally representative. Nevertheless, our results should prove to be promising findings for the promotion of a mass vaccination campaign in Vietnam.

In many countries, hesitancy and misinformation regarding vaccines and vaccination programs pose substantial obstacles to achieving herd immunity [13],[14]. Cornwall W. (2020) identified that spreading popular misinformation across multiple communication channels could have a considerable effect on the acceptance rates of a COVID-19 vaccine [15]. Misinformation can even be promulgated by scientific journals that lack a rigorous editing process [16]. Projects researching COVID-19 vaccine acceptance rate must take careful discretion in their usage of related sources [17]. Our findings showed that nearly all study respondents had been attentive towards COVID-19 vaccine related information. About 99% of them looked for vaccine information through at least one of many various communication channels. On one hand, this statistic is encouraging of increased vaccine literacy amongst Vietnamese citizens. On the other hand, misinformation could turn a number of these information seekers against the prospect of vaccination [15]. Though around 46% of respondents reported that they got information about the COVID-19 pandemic and vaccine from healthcare professionals, a larger two-thirds of all respondents reported professionals to be the most reliable information source. Access to and trust in information sources of respondents in our study are familiar with some other studies in Jordan and the United States [17],[18]. This is a good suggestion for enhancing the participation of healthcare staff in providing vaccine information to the general population.

Another interesting finding in our survey is a high proportion of WTP for COVID-19 vaccine among respondents (70.4%). This statistic corresponds with a proportion of 30% of respondents who had concerns about the cost of vaccines. In fact, the COVID-19 vaccination in Vietnam is cost-free for priority groups, which includes teachers [7]. High levels of WTP strengthen our belief in nationwide vaccine acceptance. High levels of belief in the safety and efficacy of the vaccine likely also correlate with elevated acceptance levels. Indeed, Quinn S.C. (2013) identified that public confidence in regulatory agency reports of vaccine safety and effectiveness will be crucial in improving citizens' confidence in vaccination [19]. It is fair to say that the Vietnamese government was successful in swaying public opinion in favor of their vaccine campaign; government communication campaigns should remain regular in order to continue bolstering vaccine acceptance rates.

In our survey, determinants of COVID-19 vaccine acceptance were identified from multivariate logistic regression. Per our study, older participants were found to be more likely to accept COVID-19 vaccines, similar to many previous studies [10],[17],[20]. Previous studies have been inconsistent in determining gender's role as a determinant of vaccine acceptance. Malik AA et al. (2020) and Di Gennaro F et al. (2021) indicated that males were more likely to accept the vaccine [17],[21], while Al-Mohaithef M and Padhi BK (2020) and Jeffrey V. Lazarus et al. (2021) found higher acceptance rates among females [10],[20]. A study showed that female school administrators expressed higher levels of fear towards COVID-19 than their male peers, which could explain the increased rates of vaccine acceptance among females [22]. These contrasting results could be caused by design differences between the study samples; nonetheless, it remains clear that gender is a significant determinant of vaccine acceptance. A systematic review and meta-analysis of Robinson E et al. (2021) showed no evidence linking vaccine acceptance rates and the presence of chronic health conditions in respondents [8]. However, our survey found that respondents with chronic disease(s) were less likely to accept the vaccine. Our result is also similar to that of a study conducted by Daly M. and Robinson E. in the United States in 2021 [23]. The fear of adverse events post-vaccination may contribute to decreased vaccine acceptance in populations with chronic diseases. However, people with chronic diseases are one of the priority groups for vaccination in Vietnam. So, future communication campaigns for vaccine support should take into account findings in this survey to produce more concrete and demographic-targeted communication messages.

Moreover, respondents who were doubtful about information regarding the safety and efficacy of COVID-19 vaccines were less likely to accept the vaccination than those who expressed no doubt. Again, this confirmed the idea that confidence in vaccine safety is a crucial determinant for their acceptance [19]. Exposure to misinformation about COVID-19 and public concern over the safety of vaccines were both identified as predictors of decline in public willingness to get vaccinated [23],[24]. This evidence suggests that future behavior-change communication campaigns related to the COVID-19 vaccine support should use the Health Belief Model to create a framework for understanding social and personal factors that may impact a person's intention to get vaccinated [25].

### 4.2. Limitations

Respondents in our survey were not representative of all teachers from kindergarten to high schools in Vietnam. Participants were more likely to have been those particularly concerned about COVID-19 and/or those hoping to impact vaccine-related research. This may have led to a study sample of people with greater knowledge about the virus; naturally, this could have also led to inflated levels of vaccine acceptance from our respondents. However, our findings about determinants of COVID-19 vaccine acceptance are reliable because of the size and demographic diversity of our sample. These findings should be used to improve future communication campaigns - not only for teachers but also for the whole population. Additionally, future research should focus on more social determinants around vaccine acceptance and how to deal with related obstacles as future vaccine booster doses are rolled out.

## 5. Conclusions

Intentions to be vaccinated against COVID-19 were high among teachers from kindergarten to high school in Vietnam. This is a promising indicator for high coverage among this priority group for vaccination. Vaccine acceptance changes in relation to different personal factors, including age, gender, current health conditions, and belief in the safety and efficacy of COVID-19 vaccines. Future communication campaigns should consider these determinants to achieve higher levels of vaccine acceptance.
